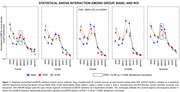# Abnormalities of Cortical Neural Synchronization Mechanisms in Patients With Dementia Due to Parkinson’s and Sintomatic Huntington’s Diseases

**DOI:** 10.1002/alz.090670

**Published:** 2025-01-03

**Authors:** Giuseppe Noce, Claudio Del Percio, Roberta Lizio, Susanna Lopez, Dharmendra Jakhar, Bahar Güntekin, Görsev Yener, John‐Paul Taylor, Marina De Tommaso, Marianna Delussi, Claudio Babiloni

**Affiliations:** ^1^ IRCCS Synlab SDN, Naples Italy; ^2^ Sapienza University of Rome, Rome, Rome Italy; ^3^ Sapienza University of Rome, Rome Italy; ^4^ Istanbul Medipol University, Istanbul Turkey; ^5^ Izmir University of Economics, Faculty of Medicine, Balçova, Izmir Turkey; ^6^ Newcastle University, Newcastle upon Tyne United Kingdom; ^7^ University of Bari Aldo Moro, Bari Italy

## Abstract

**Background:**

Parkinson’s disease and Huntington’s disease are both neurodegenerative conditions involving the basal ganglia area of the brain. Both conditions can cause symptoms that affect movement. Cognitive decline or dementia can also occur in both. Resting state EEG (rsEEG) rhythms reflect neurophysiological mechanisms and operational functions related to the fluctuation of brain arousal and quiet vigilance in humans. The hypothesis was that rsEEG sources may be more abnormal in Huntington’s disease patients in symptomatic stage (S‐HD) than patients with dementia due to Parkinson’s disease.

**Method:**

Clinical and rsEEG datasets in 16 PDD, 18 S‐HD, and 25 matched cognitively unimpaired (Nold) participants ‐ matched as demography, education, and gender ‐ were taken from an international archive. The eLORETA freeware was used to estimate cortical rsEEG sources at delta, theta, alpha1, alpha2, alpha3, beta1, beta2, and gamma frequency bands.

**Result:**

Results showed lower amplitude of the posterior alpha activities and higher amplitude of widespread low frequencies bands (i.e., delta and theta) in the PDD and S‐HD groups than in the Healthy group. As compared to the PDD group, the S‐HD showed greater reductions in the rsEEG alpha 2 rhythms in the frontal and temporal regions (see Figure 1).

**Conclusion:**

These results suggest that cortical sources of rsEEG rhythms might reflect different abnormalities of the core neurophysiological mechanisms underlying brain arousal in quiet wakefulness and low vigilance in PDD, and S‐HD patients. The mentioned rsEEG markers might be clinically useful in the disease staging, monitoring over time, and drug discovery.